# Effects of Floral Scents and Their Dietary Experiences on the Feeding Preference in the Blowfly, *Phormia regina*

**DOI:** 10.3389/fnint.2015.00059

**Published:** 2015-12-01

**Authors:** Toru Maeda, Miwako Tamotsu, Ryohei Yamaoka, Mamiko Ozaki

**Affiliations:** ^1^Department of Biology, Graduate School of Science, Kobe UniversityKobe, Japan; ^2^Department of Applied Biology, School of Science and Technology, Kyoto Institute of TechnologyKyoto, Japan

**Keywords:** floral scent, feeding preference, appetite, olfaction, taste, experience, fly

## Abstract

The flowers of different plant species have diverse scents with varied chemical compositions. Hence, every floral scent does not uniformly affect insect feeding preferences. The blowfly, *Phormia regina*, is a nectar feeder, and when a fly feeds on flower nectar, its olfactory organs, antennae, and maxillary palps are exposed to the scent. Generally, feeding preference is influenced by food flavor, which relies on both taste and odor. Therefore, the flies perceive the sweet taste of nectar and the particular scent of the flower simultaneously, and this olfactory information affects their feeding preference. Here, we show that the floral scents of 50 plant species have various effects on their sucrose feeding motivation, which was evaluated using the proboscis extension reflex (PER). Those floral scents were first categorized into three groups, based on their effects on the PER threshold sucrose concentration, which indicates whether a fly innately dislikes, ignores, or likes the target scent. Moreover, memory of olfactory experience with those floral scents during sugar feeding influenced the PER threshold. After feeding on sucrose solutions flavored with floral scents for 5 days, the scents did not consistently show the previously observed effects. Considering such empirical effects of scents on the PER threshold, we categorized the effects of the 50 tested floral scents on feeding preference into 16 of all possible 27 theoretical types. We then conducted the same experiments with flies whose antennae or maxillary palps were ablated prior to PER test in a fly group naïve to floral scents and prior to the olfactory experience during sugar feeding in the other fly group in order to test how these organs were involved in the effect of the floral scent. The results suggested that olfactory inputs through these organs play different roles in forming or modifying feeding preferences. Thus, our study contributes to an understanding of underlying mechanisms associated with the convergent processing of olfactory inputs with taste information, which affects feeding preference or appetite.

## Introduction

Flowering plants and their pollinators have frequently been studied as examples of mutualistic associations. Many plants produce flowers that act as both advertisements to attract pollinators and as reproductive organs. The pollinators must perceive the floral advertisements, with the expectation of receiving palatable rewards such as nectar, pollen, etc. (Kevan and Baker, 1983; Raguso and Willis, 2003; Fenster et al., 2004; Schäffler et al., 2012). Floral scent is a particularly important trait for pollinators, because their behaviors are driven by scented nectar (Jürgens, 2004; Dobson, 2006; Raguso, 2008; Klahre et al., 2011; Riffell et al., 2013; Byers et al., 2014). However, there are few studies that are focused on the relationships between flowers and insects via appetitive or nonappetitive effects of floral scents.

The blowfly, *Phormia regina*, visits flowers as a pollinator, and this species has historically been used in many physiological studies of gustation and feeding (Dethier, 1976; Devaud, 2003; Ozaki et al., 2003; Nisimura et al., 2005; Murata et al., 2006; Nakamura and Ozaki, 2009; Maeda et al., 2014). Regarding olfaction, the fruit fly (*Drosophila melanogaster*) has been used more prevalently for research in this field (de Bruyne et al., 1999, 2001; Vosshall, 2000, 2001; Hallem et al., 2004; Ishimoto and Tanimura, 2004; Davis, 2005; Laissue and Vosshall, 2008; Masek and Scott, 2010; Gruber et al., 2013; Kain and Dahanukar, 2015; Kirkhart and Scott, 2015; Linford et al., 2015). Nevertheless, in order to investigate relationships between floral scents and feeding preferences in insects, we concluded that a nectar feeder, *P. regina*, is more suitable than *D. melanogaster*, which prefers fermentation odors. The honeybee, *Apis mellifera* is a representative nectar feeder that has been used in many olfactory learning studies, most of which focus on learning to associate an innately neutral odor with a sweet taste reward (Bitterman et al., 1983; Hammer and Menzel, 1995; Menzel et al., 2001; Scheiner et al., 2004; Giurfa and Sandoz, 2012; Perry and Barron, 2013). However, it might be difficult to prepare honeybee individuals that are naïve to floral scents, because honeybees usually feed on scented nectar in fields and hives. However, we can prepare *P. regina* individuals that are naïve to floral scents, and we can adopt other experimental approaches to test the dietary conditioning effects of various floral scents compared to classical associative conditioning to neutral odors.

Odorants were previously classified as attractants or repellents in *D. melanogaster* studies (Ayyub et al., 1990; Devaud, 2003; Majetic et al., 2009), but these are not necessarily functionally equivalent to appetitive or nonappetitive ones. Appetite, which can be affected by food flavor, is usually measured by the amount of food ingested. However, in the present study of feeding preference with *P. regina*, we used the proboscis extension reflex (PER) threshold as an appetite measure (Nisimura et al., 2005). Flies extend their proboscis when contact chemosensilla on their legs or labella detect sugar above a certain concentration threshold. The PER threshold concentration of sugar is not always constant, but is affected by prior experiences or learning. Moreover, it depends on the daily food concentration of sugar in *P. regina* (Yano et al., 1986) and starvation in *D. melanogaster* (Nishimura et al., 2012). In *P. regina*, PER can also be conditioned to salt stimulation across sensory modalities using saltiness-sweetness-associative learning (Akahane and Amakawa, 1983), and it can be inhibited by sweetness-bitterness-associative learning in *D. melanogaster* (DeJianne et al., 1985; Brigui et al., 1990).

Thus, the feeding preference of the tested odor is evaluated by the shift in the sucrose concentration-PER curve. When the PER threshold is increased by a nonappetitive odor or decreased by an appetitive odor, the curve shifts to the right or left, respectively. Using this method in *P. regina*, Maeda et al. (2014) demonstrated that the olfactory input of an appetitive 1-octen-3-ol odor decreased the PER threshold sucrose concentration via maxillary palps instead of antennae. Shiraiwa (2008) also reported behavioral evidence in *D. melanogaster* indicating that some odorants detected by the maxillary palps enhance phagostimulative taste. These studies suggested a close integration of taste and olfactory information in the brain. Moreover, Nisimura et al. (2005) showed in the blowfly *Phormia regina* that feeding threshold to sucrose increased in the presence of the odor of D-limonene and decreased in the presence of the odor of dithiothreitol. When fed with sucrose scented with D-limonene for 5 days after emergence, flies showed subsequent decreased appetite to plain sucrose, whereas when they were fed with sucrose scented by dithiothreitol they showed increased appetite. However, mushroom body-ablated flies did not show these appetite changes. This suggests that mushroom body, the learning, and memory center of the insect brain, is necessary for the flies to apply previous experiences of food flavors to appetitive learning behaviors. Thus, it is considered that odorants can be classified as nonappetitive, neutral, or appetitive, and that some affect the appetite in an olfactory organ-dependent manner. Prior experiences of feeding on sucrose, when scented with nonappetitive and appetitive odors, decreased, and increased the appetite to plain sucrose, respectively, as if the plain sucrose solution used for the subsequent PER test was scented with those odors.

Using 50 plant species in the present study, we tested whole bouquets of every floral scent, instead of each single component, in both non-experienced and experienced blowflies, *P. regina*. Moreover, we conducted experiments that considered the differing roles of olfactory inputs between two olfactory organs, antennae and maxillary palps.

## Materials and methods

### Flies

Blowflies, *P. regina*, were reared in our laboratory under a 12 h light-12 h dark cycle at 22 ± 2°C. Larvae were fed chicken livers and yeast bait (Oriental Yeast, Japan). Water and 100 mM sucrose solution were provided to adults in separate cups. In order to harvest eggs, flies, older than 7 days after emergence, were reared in a separate cage, and were provided with water and sucrose plus chicken liver. Egg masses laid on chicken livers were collected every morning.

### Experimental paradigm for olfactory experience during sugar feeding

An adult fly population derived from the same egg mass was divided into two groups within a day after emergence, and was reared in separate plastic cages (22 × 15 × 13 cm^3^) under different dietary conditions (Nisimura et al., 2005). One group (experienced group) of flies was provided with water and 100 mM sucrose solution, scented with a floral scent, on a special meal stage for 5 days. On a double-bottomed plastic meal stage (75 mm diameter; 35 mm height), a cotton ball soaked with sucrose solution was placed in the upper dish, which was mostly airtight with the exception of pinhole openings at the edge, and a cluster of the flowers was placed in the lower compartment. Therefore, when a fly visited the meal stage and extended its proboscis to feed on sucrose, it would be simultaneously stimulated with the taste of sugar and the floral scent. When we investigated the D-limonene odor instead of floral scents, we set a cotton ball soaked with 1 mL D-limonene on the aluminum foil in the lower compartment. As a control group (non-experienced group), a second population, derived from the same batch, was provided with water and 100 mM sucrose on a meal stage with the same shape but without an odor source in the lower compartment for 5 days.

### Ablation of antennae or maxillary palps

The adults within a day after emergence or 8-day-old adults, which were provided for according to the experimental paradigm mentioned above, were anesthetized on ice for approximately 10 min. Using micro-scissors, both the antennae or the both maxillary palps of the anesthetized flies were entirely removed under a stereomicroscope (SZX-9, Olympus, Tokyo, Japan). An interval for about 30 min was taken between ablation of antennae or maxillary palps and PER test.

### PER test for appetite measurement

**Figure 1:**
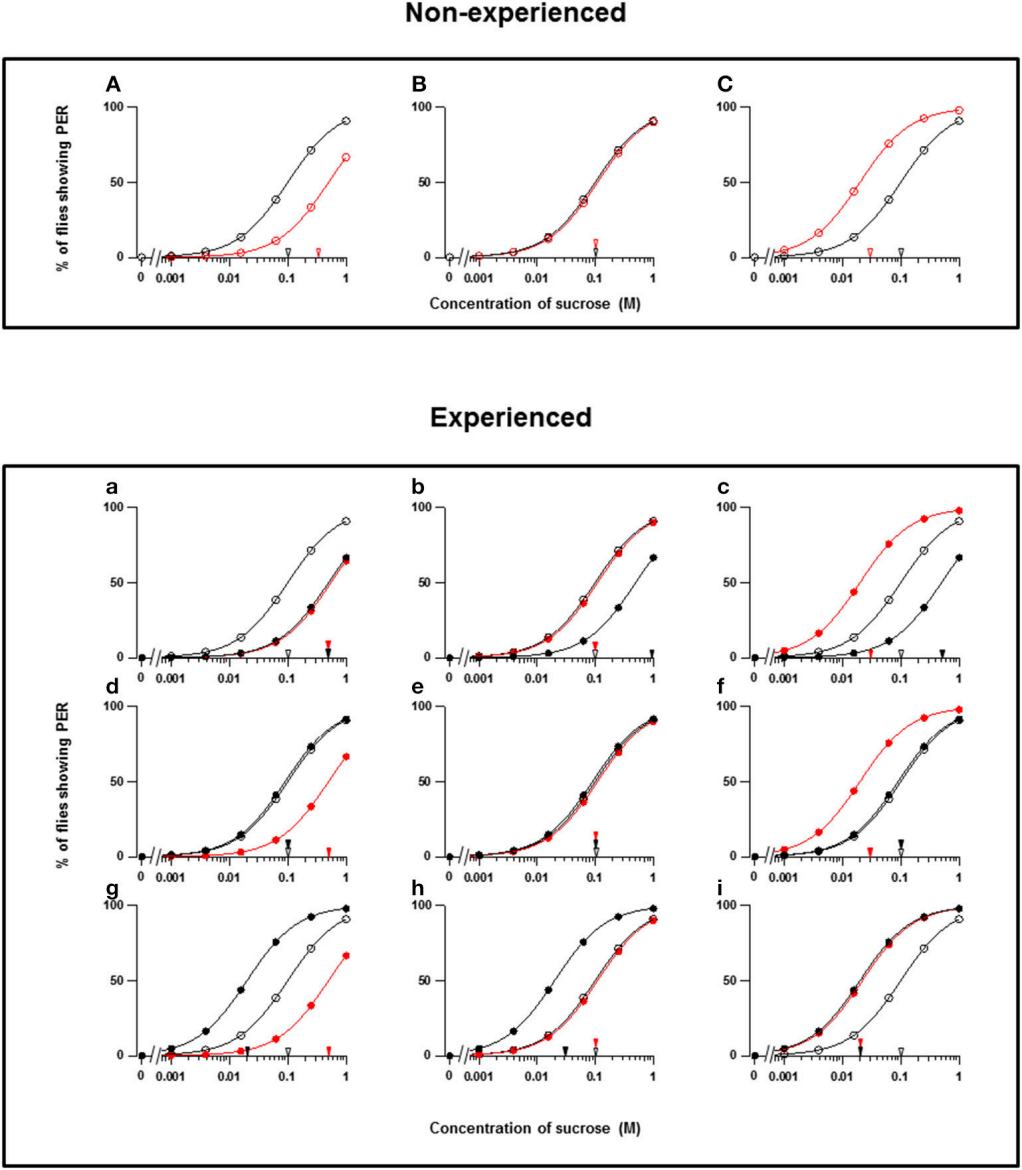
**Theoretical types of fly appetite change**. **(A–C)** Three possible effects of floral scents on the sucrose concentration-PER curves in non-experienced flies. **(a–i)** Nine possible effects of floral scents on the sucrose concentration-PER curves in experienced flies. The sucrose concentration-PER curves in the absence (black closed circles) and presence (red closed circles) of a scent are drawn. The black and red closed arrowheads indicate the mean PER threshold of sucrose concentrations in the absence and presence of scents, respectively. The concentration-PER curves in the absence of scent in non-experienced flies are drawn with open circles, and the mean PER concentration is indicated by open arrowheads. For examples, the PER curves are drawn, so that the mean threshold is increased from 0.1 to 0.5 M or decreased to 0.02 M according to respective appetite change.

In the present study, the term “appetite” was used to indicate the motivation for feeding, which we evaluated using the PER threshold as a necessary prerequisite to feeding. Decreased and increased appetites in flies indicated high and low PER thresholds to sucrose, respectively. For the PER test, we obtained the thresholds in individual test flies of 8-days-old and compared them between populations and conditions. Prior to the PER test, 20 individuals including both sexes were randomly chosen from each group and starved for 24–36 h, being provided by water, and were immobilized by securing the wings with aluminum clothespins. Before the PER test, flies were provided with water to satiation. Stimulus solutions for the PER test included 11 steps of sucrose concentrations (prepared by two-fold serial dilutions with distilled water) or five steps (prepared by four-fold serial dilutions with distilled water), and both preparations started at a 1 M-sucrose concentration. The labellar contact chemosensilla of flies were carefully stimulated by hand with each sucrose concentration in a wide-mouth 200 mL pipette tip, beginning with the lowest concentration, so that flies would not ingest stimulus solutions. If necessary, this stimulation step was conducted under a stereomicroscope (SZX-9, Olympus, Tokyo, Japan). We then examined the effects of the presence of floral scents, and the PER test was performed with the odor source set approximately 2 cm away from the fly. The sucrose concentration to which the fly first fully extended its proboscis was defined as the PER threshold at the individual level. Thus, the classification (see Figure 1) was determined so that the statistically examined differences in individual PER thresholds defined all concentration-PER curve shifts (*p* > 0.05 for non-significant shifts or *p* < 0.05 for significant shifts, Mann-Whitney U test; *n* = 20). The PER value for each fly was plotted against the sucrose concentration, and the mean threshold was determined as half the maximum sucrose concentration that induced PER in 50% of the test flies. The statistical test for the mean threshold (Mann-Whitney U test) was not done except for the data of **Figure 5I**, which was obtained from five sets of PER tests using 20 flies each. When the sucrose concentration-PER curve shifted to the left in the presence of a scent and the mean PER threshold decreased, the fly appetite was increased by the scent and vice versa. We examined each floral scent twice during two seasonal rounds in different years, and we reported the results of 50 floral scents and their effects on appetite, which were qualitatively classified into the same types.

### Floral scent analysis

The flowers used in our behavioral experiments were purchased or field-collected, and a coupled gas chromatography-mass spectrometer (GC-MS) was used for floral scent analyses. The freshly cut flowers were divided into appropriate clusters with 3–10 bloomed flowers (Number of flowers was dependent on the size of flowers or plant species), and the cut-ends were buried in a small, wet cotton ball that was tightly covered with aluminum foil. The flower cluster of every species was placed in a tightly closed polyethylene bag, and was connected to glass tubes for both air-drawing and odor-adsorption. A continuous charcoal filtered air stream (200 mL air·min^−1^) was blown into the bag by a vacuum pump, and the bag was connected to the odor-adsorbing tube (3 mm inner diameter, 6 mm outer diameter, 160 mm length), in which 60 mg of TENAX^TA^ column packing (Shimadzu Corporation, Kyoto, Japan) was packed between clean glass wool plugs. Volatiles were collected overnight at room temperature, and 2 mL diethyl ether was then poured into the odor adsorbing tube and recovered with desorbed volatiles. To condense the recovered eluent, an evaporator was used to evaporate the diethyl ether. Thus, the volatile extraction was repeated twice during blooming period of each species, except in cases of too short blooming periods. For GC-MS analyses, collected volatiles were dissolved in 40–100 μL *n*-hexane, and 2 μL of the resulting solutions were injected into a GCMS-QP5000 (splitless mode, 30 s, injector temperature 230°C; Shimadzu Cooperation). The GC equipment, with fused silica capillary columns (30 m × 0.25 mm, d.f. = 0.25 μm) and DW-wax (J&W Scientific, Folsom, CA, USA), was operated in the electron impact ionization mode. Helium was used during the mobile phase at an average linear flow rate of 35 cm·s^−1^. The GC oven temperature for both columns was programmed as follows: an initial 5 min hold at 40°C, an increase to 180°C at 5°C·min^−1^, and a subsequent increase to 200°C at 10°C·min^−1^ followed by a 10 min hold.

### Ethical approval

Our animal researches have to be performed in accordance with the relevant guidelines for ethical approval from the review committee on animal experiments in Rokkodai of Kobe University. However, this manuscript is exempt from ethics committee approval, because the blowfly, our material, is a lower invertebrate.

## Results

### Feeding preference and its modification by floral scents

Throughout our experiments, we conducted PER tests with a sucrose concentration series in both the absence and presence of a floral scent, and we then compared the PER thresholds. When the threshold was increased, unchanged, or decreased by a scent, the fly appetite was designated as decreased, unchanged, or increased, respectively. These three types of appetite change in non-experienced flies were then classified as A, B, and C, respectively (Figures 1A–C). We not only conducted the same PER test in non-experienced flies in order to investigate innate feeding preference of the tested scent, but we also tested experienced flies to investigate feeding preference modifications during olfactory experiences with the tested scents. Thus, the classification (a–i) of appetite changes in experienced flies was more complicated (Figures 1a–i). Consequently, every tested plant listed in Table 1 is classified by the combined effect of its scent on the appetite change in both non-experienced and experienced flies. In the tables, the top three components of each floral scent and the percentage of each are shown, but exclusively common components within a type are not clearly identified.

**Table 1:** **Chemical analyses of floral scents**.

**Plant species**	**Major scent components**	**%**	**Appetite change type**
*Citrus natsudaidai*	linalool	80.10	Aa
	β-myrcene	6.34	
	nerolidol	4.59	
*Heliotropium arborescens*	benzaldehyde	68.15	Aa
	2-ethyl-1-hexanol^*^	17.67	
	unidentified	2.79	
*Brassica napus*	2-ethyl-1-hexanol^*^	20.61	Ab
	benzeneacetoaldehyde	18.84	
	m-xylene	3.75	
*Daphne odora*	β-cis-ocimene	48.03	Ab
	β-trans-ocimene	41.07	
	citronellol	4.41	
*Spiraea thunbergii*	unidentified	12.60	Ab
	unidentified	11.04	
	1,2-/1,3-dimethylbenzene	9.03	
*Erigeron annuus*	p-xylene	40.48	Ae
	2-ethyl-1-hexanol^*^	14.54	
	methyl salicylate	9.70	
*Ligustrum japonicum*	m-xylene	57.2	Ae
	toluene	12.82	
	o-xylene	10.79	
*Wisteria floribunda*	trans-ocimene	62.13	Ae
	unidentified	13.73	
	benzylacetate	6.85	
*Limonium sinuatum*	p-xylene	30.34	Af
	trimethyl benzene	23.20	
	m-xylene	17.44	
*Narcissus tazetta*	limonene	76.88	Ag
	2-ethyl-1-hexanol^*^	12.22	
	dioxane	4.17	
*Ammi majus*	β-sesquiphellandrene	32.99	Ba
	unidentified	15.74	
	2-ethyl-1-hexanol^*^	9.61	
*Astilbe xarendsii*	p-xylene	62.36	Ba
	o-xylene	10.18	
	toluene	9.40	
*Dianthus superbus*	2-ethyl-1-hexanol^*^	49.83	Ba
	p-xylene	17.42	
	methyl salicylate	8.25	
*Hyacinthus orientalis*	β-trans-ocimene	41.16	Ba
	benzyl acetate	34.03	
	β-myrcene	9.60	
*Lavandula angustifola*	2-ethyl-1-hexanol^*^	51.93	Ba
	o-xylene	16.35	
	methyl salicylate	8.6	
*Mirabilis jalapa*	2-ethyl-1-hexanol^*^	27.01	Ba
	limonene	23.33	
	p-xylene	17.59	
*Jasminum polianthum*	limonene	30.13	Bb
	β-linalool	13.89	
	m-xylene	9.35	
*Lobularia maritima*	1-dococene	40.12	Bb
	2-ethyl-1-hexanol^*^	17.63	
	benzothiazole	5.37	
*Matthiola incana*	β-farnesene	65.52	Bb
	2-methoxy-3methyl-(2-propenyl) phenol	24.82	
	methyl eugenol	3.16	
*Chamelaucium uncinatum*	2-ethyl-1-hexanol^*^	46.05	Be
	γ-tepinene	10.25	
	m-xylene	8.85	
*Cosmos bipinnatus*	β-trans-ocimene	53.59	Be
	1,3,8 p-menthatriene	35.09	
	sabinene	4.81	
*Freesia refracta*	linalool	69.58	Be
	o-xylene	21.68	
	2-ethyl-1-hexanol^*^	4.67	
*Jasminum sambac*	not analyzed		Be
*Quercus serrata*	p-xylene	30.87	Be
	geranyl nitrile	18.92	
	cis-3-hexenol	10.06	
*Rhaphiolepis umbellata*	p-xylene	36.12	Be
	methyl salicylate	16.54	
	2-ethyl-1-hexanol^*^	10.99	
*Rhododendron pulchrum*	m-xylene	39.83	Be
	2-ethyl-1-hexanol^*^	18.41	
	methyl salicylate	10.56	
*Solidago altissima*	sabinene	32.56	Be
	α-pinene	27.53	
	β-myrcene	20.95	
*Abelia grandiflora*	2-ethyl-1-hexanol^*^	33.13	Bf
	benzakdehyde	29.82	
	non-anal	7.03	
*Gradenia jasminoides*	ocimene	51.28	Bf
	β-linalool	24.65	
	α-farnesene	11.87	
*Duranta repens*	limonene	32.93	Bi
	2-ethyl-1-hexanol^*^	24.96	
	m-xylene	24.51	
*Elaeagnus umbellata*	p-cresol methyl ester	28.45	Bi
	1,4 dimethoxy benzene	20.92	
	m-xylene	16.79	
*Mahonia japonica*	trans-ocimene	68.07	Bi
	benzaldehyde	9.48	
	cis-ocimene	7.46	
*Nothoscordum striatum*	m-xylene	49.19	Bi
	2-methoxy-1-propanol	21.62	
	methyl salicylate	9.06	
*Osmanthus fragrans*	linalool	24.07	Bi
	geraniol	17.84	
	dodecane	6.10	
*Rosa multiflora*	germacrene D	50.23	Ca
	2-ethyl-1-hexanol^*^	16.35	
	4-ethl-1-hexanol	16.35	
*Luclia pinceana*	unidentified	43.49	Cb
	unidentified	5.83	
	3-phenyl-3-butene-3-one	5.50	
*Farfugium japonicum*	unidentified	59.61	Ce
	2-ethyl-1-hexanol^*^	16.66	
	1-undecene	12.80	
*Fatsia japonica*	2-ethyl-1hexanol^*^	37.83	Ce
	p-xylene	15.43	
	D-limonene	14.08	
*Hydrangea macrophylla*	o-xylene	37.20	Ce
	methyl salicylate	13.83	
	2-ethyl-1-hexanol^*^	12.25	
*Spriarea betulifola*	not analyzed		Ce
*Trifolium repens*	caryophyllene	14.67	Ce
	α-farnesene	12.63	
	N-phenyl formamide	12.58	
*Bignonia capreolata*	p-xylene	27.32	Cf
	2-ethyl 1-hexanol^*^	14.84	
	1-methoxy 2-propanol	8.86	
*Chrysanthemum morifolium*	2,7,7-trimethyl bicyclo (3,1,1,)-hept 2-en 6-one	20.34	Cf
	β-farnesene (E)	20.33	
	2-ethyl 1-hexanol	11.40	
*Hedera rhombea*	2-ethyl 1-hexanol	19.09	Cf
	limonene	15.32	
	m-xylene	12.11	
*Tagetes patula*	cis-ocimene	27.58	Cf
	2-ethyl 1-hexanol^*^	23.24	
	caryophyllene	13.53	
*Viola odorata*	2-ethyl-1-hexanol^*^	53.54	Cf
	p-xylene or o-xylene	12.24	
	unidentified	4.52	
*Verbena hortensis*	epoxylinalool	34.63	Ch
	2-ethyl-1-hexanol^*^	24.81	
	reduced form of epoxylinalool	16.78	
*Hedychium coronarium*	trans-ocimene	35.64	Ci
	methyl benzoate	16.65	
	methyl salicylate	8.93	
*Paederia scandens*	acetophenone	59.96	Ci
	unidentified	8.10	
	2-ethyl-1-hexanol^*^	6.43	
*Trifolium pratense*	cis-3-hexenyl acetate	22.47	Ci
	trans-ocimene	17.62	
	3-hexene-1-ol	14.21	

* *Possibly including artificial contamination from plastic parts of GC-MS equipment*.

**Figure 2:**
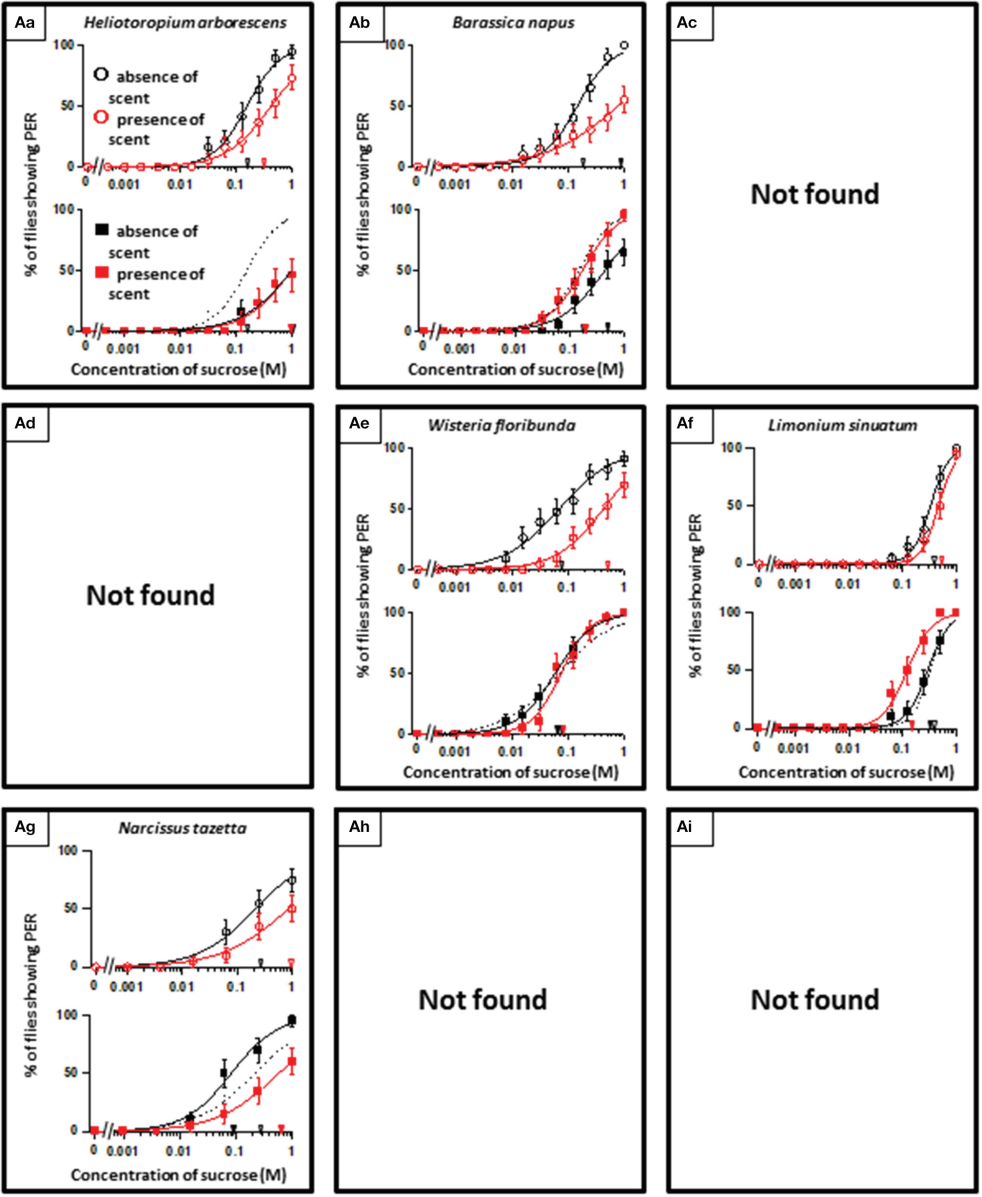
**The representative sucrose concentration-PER curves modified by innately nonappetitive floral scents**. Top of each panel in Figures 2–4: Effect of floral scent in non-experienced flies. The curves in the absence (black open circles) and presence of scent (red open symbols) are drawn. The black and red open arrowheads indicate the mean PER threshold of sucrose concentrations in the absence and presence of scent, respectively. Bottom of each panel in Figures 2–4: Effect of floral scent in experienced flies. The curves in the absence (black closed squares) and presence of scent (red closed squares) are drawn. The black and red closed arrowheads indicate the mean PER threshold of sucrose concentrations in the absence and presence of scent, respectively. Broken line indicates the curve in the absence of scent in non-experienced flies. All of the plots are presented as the averages with SEM in Figures 2–**6**.

**Figure 3:**
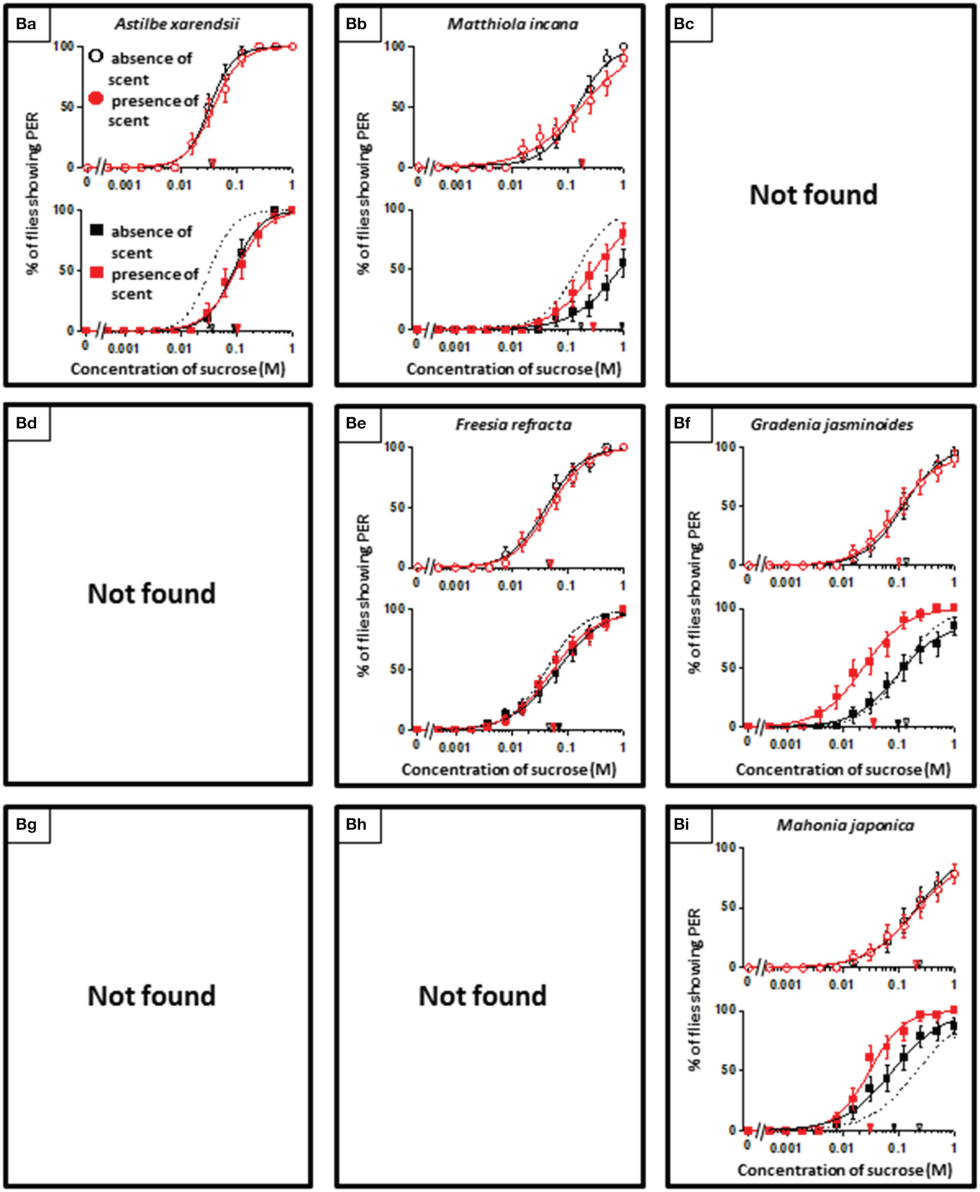
**The representative sucrose concentration-PER curves modified by innately neutral floral scents**.

**Figure 4:**
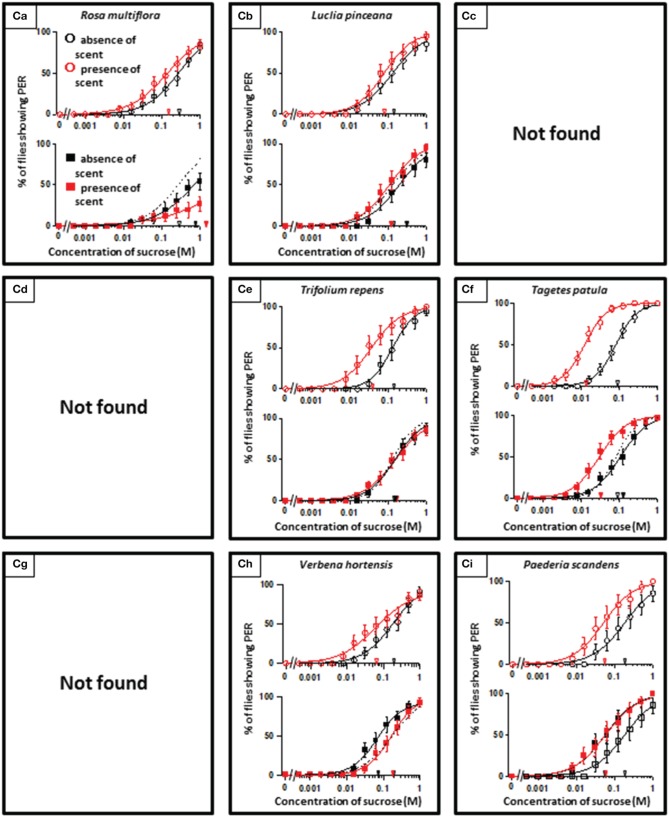
**The representative sucrose concentration-PER curves modified by innately appetitive floral scents**.

In theory, 27 types of appetite change could potentially be expected, but only 16 types (Aa, Ab, Ae, Af, Ag, Ba, Bb, Be, Bf, Bi, Ca, Cb, Ce, Cf, Ch, and Ci) appeared in our experiments. Ten species had floral scents that decreased appetite in non-experienced flies, which were divided into five types: Aa (two species); Ab (three species); Ae (three species); Af (one species); Ag (one species). Twenty four species had floral scents that have no effects on appetite in non-experienced flies, which were divided into five types: Ba (six species); Bb (three species); Be (eight species); Bf (two species); Bi (five species). Sixteen species had floral scents that increased appetite in non-experienced flies, which were divided into six types: Ca (one species); Cb (one species); Ce (five species); Cf (five species); Ch (one species); Ci (three species). Figures 2–4 show the appetite changes in the sucrose-concentration-PER curves based on the floral scents of the following representative species for each type: Aa, *H. arborescens*; Ab, *B. napus*; Ae, *W. floribunda*; Af, *L. sinuatum*; Ag, *N. tazetta*; Ba, *A. arendsii*; Bb, *M. incana*; Be, *F. refracta*; Bf, *G. jasminoides*; Bi, *M. japonica*; Ca, *R. multiflora*; Cb, *L. pinceana*; Ce, *T. repens*; Cf, *T. patula*; Ch, *V. hortensis*; and Ci, *P. scandens*.

### Effects of olfactory organ ablation on feeding preference modification

**Figure 5:**
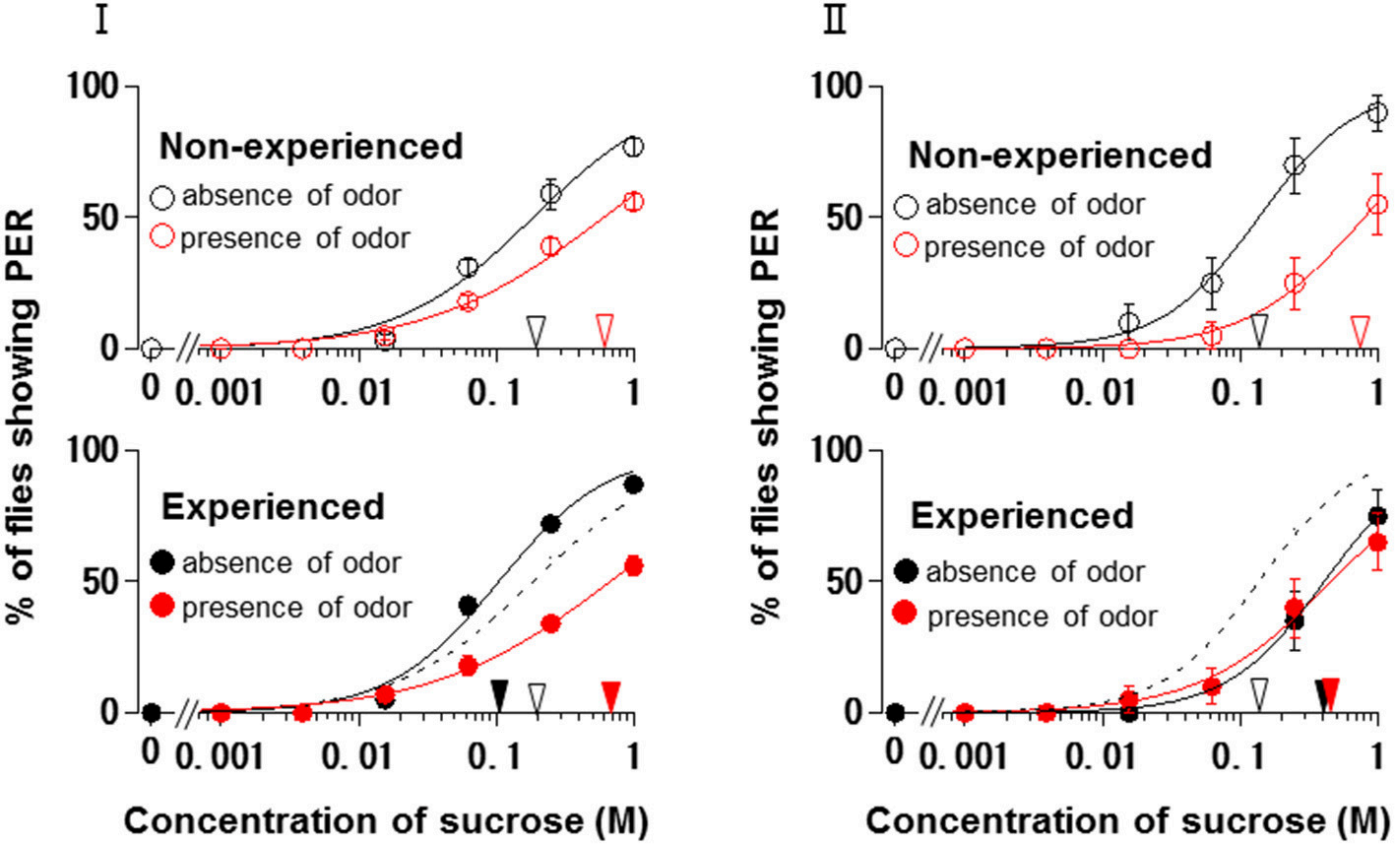
**The sucrose concentration-PER curves modified by ***Narcissus tazetta*** floral scent and its major component**. **(I)** The curves in the absence (black circles) and presence (red circles) of the *N. tazetta* scent in non-experienced (open circles) and experienced flies (closed circles). **(II)** The curves in the absence (black circles) and presence of the D-limonene odor (red circles) in non-experienced (open circles) and experienced flies (closed circles). The red and black arrowheads indicate the mean PER threshold sucrose concentrations in the absence and presence of odor; the open and closed arrowhead in non-experienced and experienced flies, respectively. Broken lines indicate the concentration-PER curves in the absence of scent in non-experienced flies.

Using *N. tazetta* typed as Ag, we examined the effects of floral scent on appetite change in groups of non-experienced and experienced flies when either the antennae or maxillary palps were ablated. The reason why we used *N. tazetta* was that the major component of its scent was found to be limonene, which has reported as an oral toxic compound to be repelled for *P. regina* (Ozaki et al., 2003; Nisimura et al., 2005). As the control experiments and in order to confirm the type of effects of its scent (Table 1, Figures 2–4), we repeatedly performed five PER tests with the intact fly groups of 20 individuals each, in which both olfactory organs were preserved. In the presence of the *N. tazetta* scent, the non-experienced flies significantly increased individual PER threshold sucrose concentrations by the presence of the floral scent (*p* < 0.001, Mann-Whitney U test; *n* = 100) (Figure 5I Top). The mean PER threshold in the presence of the floral scent (average ± standard error of means (SEM): 0.625 ± 0.216 M; *n* = 5) increased approximately three-fold compared to that in the absence of scent (0.213 ± 0.120 M; *n* = 5). There was a significant difference between these mean PER thresholds (*p* < 0.05, Mann-Whitney U test; *n* =5). When the flies had an olfactory experience with the *N. tazetta* floral scent during sugar feeding for 5 days (Figure 5I Bottom), the individual PER threshold in the absence of scent significantly decreased (*p* < 0.05, Mann-Whitney U test; *n* = 100). On the other hand, the individual PER threshold in the presence of scent significantly increased (*p* < 0.05, Mann-Whitney U test; *n* = 100). Thus, the mean PER threshold in the absence of scent (0.106 ± 0.027 M; *n* = 5) significantly decreased to half of that observed in the non-experienced flies tested in the absence of scent (0.213 ± 0.120 M; *n* = 5). However, the mean PER threshold in the presence of scent (0.781 ± 0.297 M; *n* = 5) increased approximately four-fold compared to that of the non-experienced flies tested in the absence of scent (0.213 ± 0.120 M; *n* = 5) (*p* < 0.01, Mann-Whitney U test; *n* = 5). In conclusion, the appetite change induced by the *N. tazetta* floral scent was then determined to be the Ag type in accordance with our classification (Figure 1). As the major components of the scent of *N. tazetta* was limonene (Table 1), we compared this Ag type of data on the scent of *N. tazetta* (Figure 5I) with that on D-limonene odor (Figure 5II), which was found to be classified into the Aa type.

**Figure 6:**
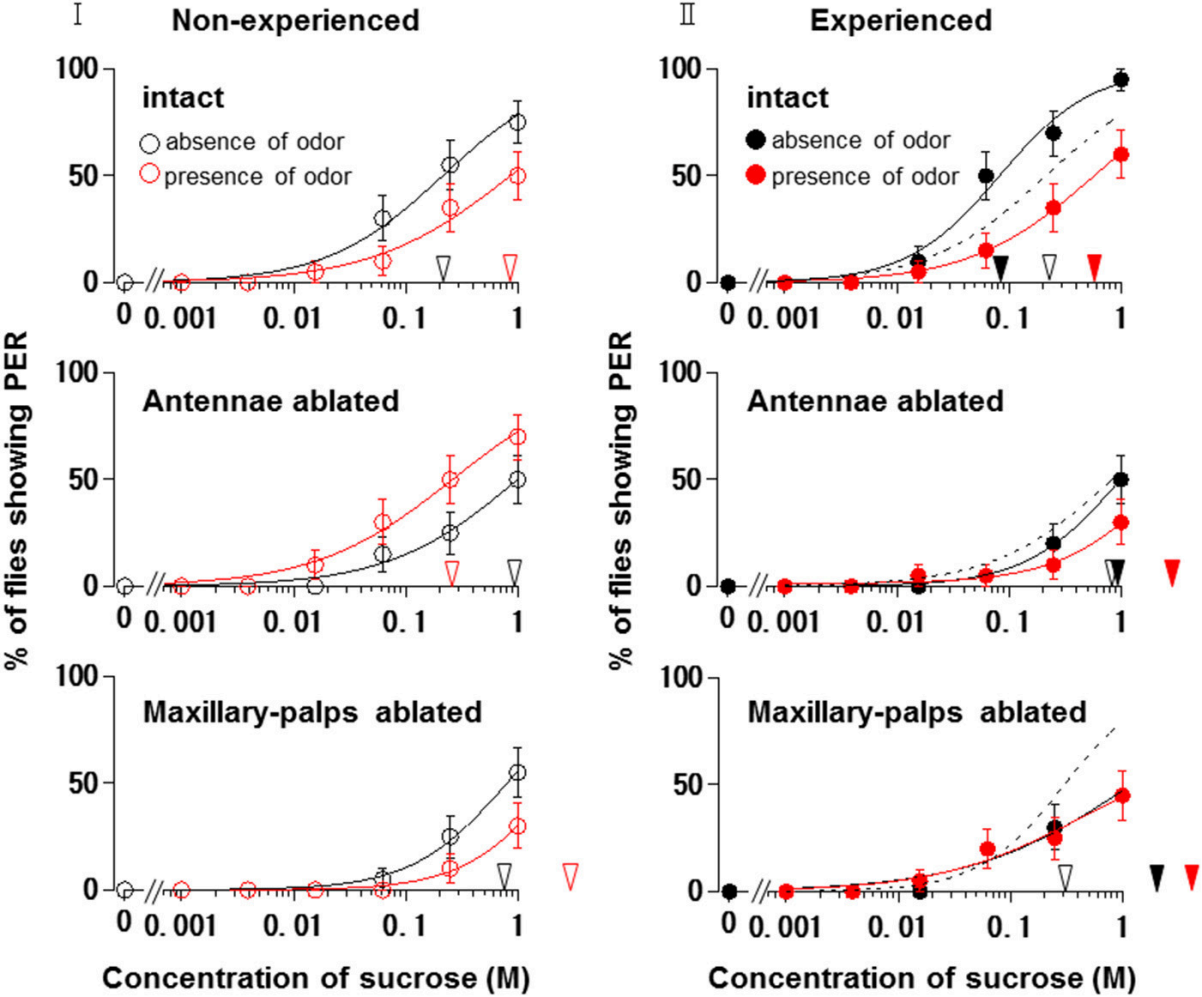
**Effects of olfactory organ ablation on the sucrose concentration-PER curves modified by ***Narcissus tazetta*** floral scent**. **(I)** Olfactory organ ablation was carried out prior to the PER tests and its effects are compared among curves in non-experienced flies (open circles); **(II)** Olfactory organ ablation was carried out prior to the olfactory experience and its effects are compared among curves in the experienced flies (closed circles). Top: The curves in the absence (black circles) and presence (red circles) of scent in the intact flies. Middle: The curves in the absence and presence of scent in the antennae-ablated flies. Bottom: The curves in the absence and presence of scent in the maxillary-palp-ablated flies. The black and red arrowheads indicate the mean PER threshold sucrose concentrations in the absence and presence of scent, respectively. Broken lines indicate the curves in the absence of scent in the non-experienced flies.

Figure 6I shows the appetite change of flies when either the antennae or maxillary palps were ablated prior to PER tests in non-experienced flies. When the antennae were ablated and the maxillary palps were preserved, fly appetites in the presence of the *N. tazetta* floral scent increased, and the individual PER decreased significantly (*p* < 0.05, Mann-Whitney U test; *n* = 20). The mean PER threshold (0.261 M) then decreased to about a quarter of that observed in the absence of scent (0.997 M). When the maxillary palps were ablated and the antennae were preserved, the fly appetites in the presence of scent decreased, and the individual PER increased significantly (*p* < 0.05, Mann-Whitney U test; *n* = 20). The mean PER threshold then increased approximately three-fold (2.166 M) compared to that observed in the absence of scent (0.800 M).

Figure 6II shows the appetite change of experienced flies when either the antennae or maxillary palps were ablated prior to the beginning of the olfactory experience with the *N. tazetta* floral scent. When the antennae were ablated and the maxillary palps were preserved, the fly appetites did not change in the absence of scent, and the individual PER threshold did not change significantly (*p* > 0.05, Mann-Whitney U test; *n* = 20). The fly appetites tended to decreased in the presence of scent, although the individual PER threshold did not significantly increase (*p* > 0.05, Mann-Whitney U test; *n* = 20). The mean PER threshold (2.629 M) increased approximately three-fold compared to that of the non-experienced flies tested in the absence of scent (0.842 M). When the maxillary palps were ablated and the antennae were preserved, the fly appetites in both the absence and presence of scent decreased, and the individual PER threshold increased significantly (*p* < 0.05 Mann-Whitney U test; *n* = 20). The mean PER thresholds in the absence (1.205 M) and presence of scent (1.770 M) increased approximately four-fold and six-fold, respectively, compared to that of the non-experienced flies tested in the absence of scent (0.303 M).

These results indicated that the appetite change type induced by the *N. tazetta* floral scent changed from Ag to Cd when the antennae were ablated and from Ag to Aa when the maxillary palps were ablated.

## Discussion

Floral scents variably affected the behavior of flower-visiting nectar feeders (Riffell et al., 2013). However, in the present study, we found that a significant number of flowers have scents that have neutral effects on fly appetites (Table 1, Figure 3). Although floral scents that are totally ineffective on appetites may differ among insect species, eight of the 50 floral scents examined here were classified as the Be type, and they had no effect on *P. regina* appetites, regardless of prior olfactory experiences. However, after olfactory experiences during sucrose feeding, innately neutral scents sometimes converted to nonappetitive (typed as Ba) or appetitive (typed as Bf or Bi) (Figure 3). Moreover, olfactory experiences with some scents decreased (typed as Ba or Bb) or increased appetite to plain sucrose (typed as Bi) (Figure 3). These phenomena indicated that feeding preference formation or modification is highly malleable and difficult to explain. However, it is unlikely that innately neutral scents cannot stimulate olfactory sensory systems at all. Otherwise, olfactory information of these scents might be blocked to access putative neural circuits for cross-modal integration with the sweet taste information of sucrose. In some cases, olfactory experiences during sucrose feeding could likely allow such olfactory information and/or associated memory to access those neural circuits by unknown mechanisms.

In other cases, when a floral scent is capable of decreasing or increasing sucrose appetites in non-experienced flies, this scent can be regarded as an unconditioning stimulus that innately increases or decreases the PER threshold sucrose concentration. After dietary conditioning with this type of scent that is innately nonappetitive or appetitive, the PER threshold sucrose concentration increased (typed as Aa) (Table 1, Figure 2) or decreased (typed as Ci) in the absence of scent (Table 1, Figure 4), respectively. Prior to our study, Nisimura et al. (2005) reported Aa and Ci appetite change types after olfactory experiences during sucrose feeding with the single odor components, D-limonene and dithiothreitol, respectively. Moreover, flies with missing mushroom bodies did not show the appetite changes observed in intact flies. This suggested that associative learning of sugar taste with nonappetitive or appetitive odors was involved in these appetite change types. However, in this context, the stimulus usage was completely different from that reported in typical olfactory associative learning, in which a sweet taste stimulus was used as a reward for neutral olfactory stimulus learning (Bitterman et al., 1983; Hammer and Menzel, 1995; Menzel et al., 2001; Scheiner et al., 2004; Giurfa and Sandoz, 2012; Perry and Barron, 2013).

On the other hand, we found the cases where olfactory experiences during sucrose feeding had no effects on appetite change. Although we found no innately appetitive cases classified as the Ad type, five kinds of floral scents increased appetites in the presence of scent regardless of olfactory experiences during sucrose feeding (typed as Cf) (Figure 4). In those cases, the flies did not learn to associate the sugar taste with the scents and the effects were typed as Ad and Cf, or learned information might not affect feeding preference for unknown reasons.

A remarkable finding in the present paper is that, regarding scented sucrose solutions, the feeding preference drastically changed after olfactory experiences. For instance, the non-experienced flies preferred plain sucrose to sucrose scented with 10 different floral scents typed as Aa, Ab, Ae, Af, and Ag. Then, experienced flies showed consistent appetite levels to sucrose that was scented with six of these floral scents (typed as Ab or Ae) compared to plain sucrose. No tested scent was typed as Ad, in which innately nonappetitive scent was still nonappetitive after olfactory experience with little effects of learning and memory. This type was appeared as the effect of D-limonene odor in the mushroom body-ablated fly (Nisimura et al., 2005). The sucrose scented with *L. sinuatum* was rather addictive (typed as Af) (Figure 2). This is the case that innately nonappetitive scent turned to be appetitive during olfactory experience in sucrose feeding. In contrast, compared to plain sucrose, non-experienced flies preferred sucrose scented with 16 kinds of floral scents typed as Ca, Cb, Ce, Cf, Ch, and Ci, but experienced flies showed consistent appetite levels to sucrose scented with seven of these floral scents (typed as Cb, Ce, or Ch). Moreover, the olfactory experience caused loss of appetite either for unscented plain sucrose or *R. multiflora*-scented sucrose (typed as Ca) (Figure 4). These phenomena cannot be explained by simple associative learning.

The floral scent of *N. tazetta* is classified as the Ag type based on its effects on appetite as previously mentioned (Table 1). However, when we repeated the PER test with the *N. tazetta* scent, the results were classified as Ag (Figure 5I). The presence of the *N. tazetta* scent decreased appetite in both non-experienced and experienced flies, while prior olfactory experience with the scent increased appetite to unscented plain sucrose. From this increased level, the appetite to scented sucrose then decreased below the original level (typed as Ag) (Figure 5I). As mentioned in the “Results” Section, the Ag type was determined for the *N. tazetta* scent using statistical tests of the PER threshold sucrose concentration.

The *N. tazetta* floral scent mainly includes limonene (Table 1), which was reported to show severe oral toxicity in *P. regina* regardless of D- or L-limonene (Ozaki et al., 2003). Therefore, the *N. tazetta* scent must be noted as a noxious odor during the olfactory experience, so the flies would then avoid sucrose scented by *N. tazetta* as well as by D-limonene (Figure 5), leading to gradual starvation. If the noxious odor was removed, the flies could recover their appetite to unscented plain sucrose. This is one of the putative explanations being considered as a feeding strategy of *P. regina* for survival (Supplement Figure 1).

When we conducted the same experiments in the antennae- or maxillary palp-ablated flies, the results were expected to give significant explanations for complicated appetite changes induced by floral scents and corresponding olfactory experiences during sucrose feeding (Supplement Figure 1). In the non-experienced flies, when the antennae were ablated, the *N. tazetta* scent perceived by the maxillary palps increased their sucrose appetite (Figure 6I, Middle). This was clearly different from the nonappetitive effect of the scent, which was demonstrated in intact flies (Figure 6I Top). On the other hand, when the maxillary palps were ablated in non-experienced flies, the *N. tazetta* scent decreased their sucrose appetite (Figure 6I Bottom), which is similar to that observed in intact flies (Figure 6I Top). These results suggested that the *N. tazetta* scent has some unknown components that can increase or decrease appetite via maxillary palps or antennae, respectively (see Supplement Figure 1I).

When either the antennae or maxillary palps were ablated prior to the olfactory experience with the *N. tazetta* scent during sucrose feeding, the experienced flies decreased appetite to sucrose scented with the *N. tazetta* scent (Figure 6II). Appetites to unscented sucrose decreased in the maxillary palp-ablated flies (Figure 6II Bottom) but not in antennae-ablated flies (Figure 6II Middle). Thus, the type of effect on appetite changed from Ag to Cd when the antennae were ablated and to Aa when the maxillary palps were ablated (Figure 6). The appetite change type in antennae-ablated flies, Cd, could be explained, if the scent via maxillary palps, which was appetitive in the non-experienced flies (Figure 6I Middle), induced loss of appetite after the olfactory experience during sucrose feeding (Figure 6II Middle) (Supplement Figure 1). Associative learning, which was previously suggested by Nisimura et al. (2005), could explain the appetite change type in the maxillary palp-ablated flies, Aa. In intact flies, the prior olfactory experience with the D-limonene odor during sucrose feeding decreased the appetite to unscented plain sucrose (typed as Aa) (Figure 5II). When the bitter taste receptor neuron was stimulated by D-limonene, *P. regina* exhibited vigorous vomiting and excretion (Ozaki et al., 2003). Therefore, a toxic substance such as D-limonene must be sensitively detected and learned by its odor to reduce phagostimulative effects of sugar taste. Consequently, D-limonene was presumed to be the most likely nonappetitive odor component of the *N. tazetta* scent and as the source of decreased appetite to unscented plain sucrose in experienced flies (via antennal input) (Supplement Figure 1II). Considering the difference between the effect of the *N. tazetta* floral scent (typed as Ag; Figure 5I) and that of the D-limonene odor (typed as Aa; Figure 5II), it was predicted that the *N. tazetta* floral scent has unknown odor components, maxillary palp input of which can block the nonappetitive memory formation or cancel the nonappetitive memory even if it was formed.

In order to fully understand the variety of floral scent effects on appetite change in nectar-feeding insects, further studies are required to examine role sharing between antennae and maxillary palps as olfactory inputs (Shiraiwa, 2008; Maeda et al., 2014). Moreover, knowledge of the neural circuit from the phagostimulative taste input to the PER expression is also needed. Recently, Kain and Dahanukar (2015) reported second-order sweet gustatory projection neurons (sGPNs) in the *Drosophila* brain, and they proposed that the antennal mechanosensory and motor center (AMMC) acts as an immediate higher-order processing center for sweet taste. Moreover, Miyazaki et al. (2015) independently discovered other sGPNs that relayed information from *Gr5a*-expressing sugar receptor neurons to distinct regions in the gnathal ganglion (GNG), and Flood et al. (2013) identified a pair of command neurons for sugar taste-induced PER. Recently, Maeda et al. (2014) showed direct interactions between maxillary olfactory and labellar gustatory neurons that explain the effect of an appetitive odor component, but many other connections are expected to be involved in feeding preference formation and its flexible modification. When the complete shape of the neural circuit for PER triggering is revealed, we will be able to identify the integration points between taste and olfactory information in the circuit.

### Conflict of interest statement

The authors declare that the research was conducted in the absence of any commercial or financial relationships that could be construed as a potential conflict of interest.
