# Supplementary material for: Effects of Floral Scents and Their Dietary Experiences on the Feeding Preference in the Blowfly, Phormia regina
Source: Front Integr Neurosci. 2015 Dec 1;9:59. doi: 10.3389/fnint.2015.00059 (PMC4664696; doi:10.3389/fnint.2015.00059)
Supplement: Supplementary file 1 [file Image1.PDF]

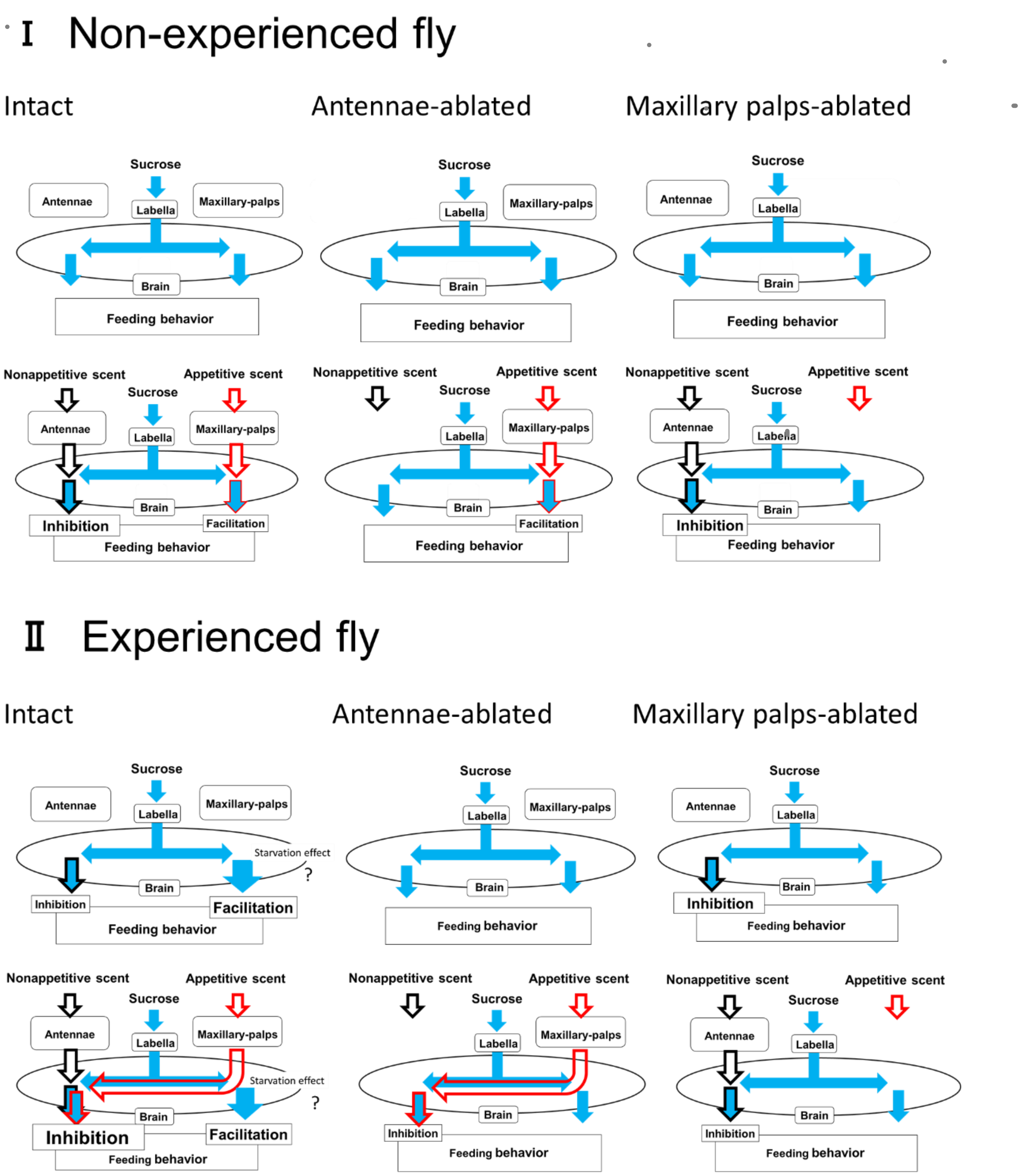

Image 1.

Model for explanation of feeding behavior modification by floral scent of *N. tazetta*.

In non-experienced (I) and experienced fly (II) in the absence (Top) and presence of scent (Bottom), gustatory information flow of sucrose taste input from the labellar chemosensilla is indicated by blue arrows, which can integrated in the brain with inhibitory and/or facilitative olfactory information derived from antennal (black open

arrows) and/or maxillary palps inputs (red open arrows). Relative strength of inhibition and facilitation of feeding behavior leading appetite decrease and increase are indicated by letter sizes of “Inhibition” and “Facilitation”, respectively. Each panel of I and II are divided the cases of intact (Left), antennae-ablated (Middle) and maxillary palps-ablated flies (Right).
